# Accuracy and Validity of a Single Inertial Measurement Unit-Based System to Determine Upper Limb Kinematics for Medically Underserved Populations

**DOI:** 10.3389/fbioe.2022.918617

**Published:** 2022-06-27

**Authors:** Charmayne Mary Lee Hughes, Bao Tran, Amir Modan, Xiaorong Zhang

**Affiliations:** ^1^ NeuroTech Lab, Health Equity Institute, San Francisco State University, San Francisco, CA, United States; ^2^ Department of Kinesiology, San Francisco State University, San Francisco, CA, United States; ^3^ School of Engineering, San Francisco State University, San Francisco, CA, United States

**Keywords:** low-and middle-income countries, stroke, rehabilitation, inertial measurement unit, kinematics

## Abstract

Stroke is one of the leading causes of death and disability worldwide, with a disproportionate burden represented by low- and middle-income countries (LMICs). To improve post-stroke outcomes in LMICs, researchers have sought to leverage emerging technologies that overcome traditional barriers associated with stroke management. One such technology, inertial measurement units (IMUs), exhibit great potential as a low-cost, portable means to evaluate and monitor patient progress during decentralized rehabilitation protocols. As such, the aim of the present study was to determine the ability of a low-cost single IMU sensor-based wearable system (named the T’ena sensor) to reliably and accurately assess movement quality and efficiency in physically and neurologically healthy adults. Upper limb movement kinematics measured by the T’ena sensor were compared to the gold standard reference system during three functional tasks, and root mean square errors, Pearson’s correlation coefficients, intraclass correlation coefficients, and the Bland Altman method were used to compare kinematic variables of interest between the two systems for absolute accuracy and equivalency. The T’ena sensor and the gold standard reference system were significantly correlated for all tasks and measures (*r* range = 0.648—0.947), although less so for the Finger to Nose task (*r* range = 0.648—0.894). Results demonstrate that single IMU systems are a valid, reliable, and objective method by which to measure movement kinematics during functional tasks. Context-appropriate enabling technologies specifically designed to address barriers to quality health services in LMICs can accelerate progress towards the United Nations Sustainable Development Goal 3.

## Introduction

According to the most recent estimates, stroke is currently the second leading cause of death (11.6% of total deaths) and the third leading cause of disability [5.7% of total disability-adjusted life-years (DALYs)] (GBD 2019 Stroke Collaborators, 2021). While stroke is a major public health issue with an increasing global economic and social burden, there are apparent variations in the geographic distribution of stroke burden (Feigin et al., 2022). Although stroke-related DALYs in high-income countries have decreased from 16.4 million to 13.1 million in the years between 2009 and 2019, that number has increased from 75.1 million to 111.0 million for low- and lower-middle income countries (LMICs) during the same time period (Feigin et al., 2022).

When comparing stroke-related deaths and disability based on World Bank income level, it has been reported that individuals residing in low-income countries are 3.6 times more likely to die from a stroke or complications, and have 3.7 times higher rate of stroke-related DALYs, when compared to individuals from high-income countries (HICs, GBD 2019 Stroke Collaborators, 2021). In addition, individuals from LMICs often exhibit poorer outcomes (Langhorne et al., 2018). For example, the INTERSTROKE study examined patient outcomes across 32 countries, and reported that individuals from LMICs were more likely to have severe or very severe strokes (52 vs. 20%) and reduced level of consciousness (54 vs. 7%) compared to their counterparts from HICs. In addition, stroke outcomes were worse for patients in LMICs, who exhibited both poorer survival (88 vs. 98%) and survival without severe disability scores (78 vs. 90%) than individuals in HICs.

Generally, individuals with stroke have impaired sensorimotor (Mahak et al., 2018; Baye et al., 2020) and psychological function (Miranda et al., 2018), limitations performing daily activities (Teasell et al., 2020), unanticipated financial burdens (Kaur et al., 2014), and reduced quality of life (Akosile et al., 2013). For example, Mahak et al. (2018) examined stroke outcomes in Chandigarh India, and reported that 63.6% of patients exhibited hemiparesis, 25.7% exhibiting difficulties performing activities of daily living (ADLs), 45.5% exhibiting difficulties in performing social activities, and 23.6% exhibiting hemiplegia. Another study conducted in Johannesburg South Africa reported that 43% of stroke patients had no or minimal reintegration into their community, and expressed particular difficulties returning to work and taking on responsibilities with their extended families (Maleka, Kusambiza-Kiingi, & Ntsiea, 2017).

Recovery from stroke often requires physical therapy aimed at improving upper limb functioning and reducing long-term disability (Pollock et al., 2014). Overall, there is evidence suggesting that physical rehabilitation strategies incorporating repetitive motor practice can facilitate neuroplasticity and brain reorganization (Veerbeek et al., 2014). However, this mode of rehabilitation requires frequent one-on-one interactions with therapists that can last for several months, and place a significant burden on nations with a shortage of trained health professionals crucial to the delivery of rehabilitation services. Individuals residing in LMICs may have to travel long distances to access the same services that residents from HICs can more easily access, which places a substantial burden on patients living in these countries, especially for individuals with low incomes, no paid time off from their jobs, physical limitations, or without access to personal transportation.

It has rightfully been suggested that the toll of stroke in LMICs can be reduced by multidisciplinary research and capacity-building, the development and promotion of evidence-based stroke prevention and intervention services, and enhancing stroke awareness among the general population (Akinyemi et al., 2021). Unfortunately, even if such transformations were to begin this year, it would take decades to close the stroke burden gap. Because the current stroke burden in LMICs necessitates urgent measures to improve post-stroke outcomes, researchers have sought to leverage emerging technologies that can help LMICs take advantage of new technologies that overcome traditional barriers associated with stroke management (Sureshkumar et al., 2016; Sarfo et al., 2018), while also skipping inefficient or more expensive healthcare methods (Jakab et al., 2018). Mobile technologies have shown some promise in the decentralized physical rehabilitation of stroke patients residing in LMICs. For example, Sarfo et al. (2018) used the 9zest Stroke Rehabilitation App to deliver a 12 week stroke exercise program to Ghanaian stroke patients in their home environment. Compared to baseline, participants exhibited lower functional impairment at both 1 month and 3 month follow-up. In addition, although 60% of patients reported major challenges with internet connectivity and the stability of the audiovisual stream, 60% of participants reported excellent satisfaction with the mobile health enabled intervention, and all participants reported they would use the system in the future.

Another enabling technology are inertial measurement units (IMUs) that exhibit great potential as a low-cost, portable means to evaluate and monitor patient progress during decentralized rehabilitation protocols (Parrington et al., 2021). IMUs have been shown to accurately measure motor function and provide information regarding the different motor components that contribute to task performance, (e.g., movement accuracy, efficiency, precision, smoothness) (Hughes et al., 2019a; Schwarz et al., 2019), with kinematic variables obtained by IMU-based system correlating with standard clinical assessments (Hughes et al., 2019b; Oubre et al., 2020; Chen et al., 2021). For example, Hughes et al. (2019a), Hughes et al. (2019b) developed a low-cost IMU-based wearable sensor specifically designed for medically underserved populations. The validity of the sensor was recently compared to a gold standard optoelectronic motion capture system (Hughes et al., 2019a), with results indicating strong positive correlations and agreement with the reference system across three tasks used to measure post-stroke arm function and impairment. The sensitivity of the sensor was then tested in Ethiopian acquired brain injured patients (Hughes et al., 2019b), with results demonstrating that the sensor is capable of deriving movement kinematics (e.g., movement time, movement smoothness, and peak velocity) that can discriminate between arm impairment levels, as measured by the Fugl-Meyer Upper Extremity Motor Assessment.

While the sensor proved to be a valid and sensitive measure of kinematic analysis, changes needed to be made to the sensor system to reduce the weight and improve the robustness of the system (Tran et al., 2022). The microcontroller, IMU, and power source were upgraded and then integrated into a single printed circuit board (PCB). These hardware modifications resulted in firmware upgrades, specifically, the original Inter-Integrated Circuit (I^2^C) synchronous communication protocol was upgraded to Serial Peripheral Interface (SPI) and the IMU’s internal First-In-First-Out (FIFO) buffer was enabled. Together, these firmware changes allowed for higher efficiency and timing precision with processing and transmission of bulk measurements. Given the considerable hardware and firmware changes, it is essential that the sensor system undergoes the revalidation process prior to clinical validation and product commercialization. As such, the aim of the present study was to determine the ability of the redesigned IMU sensor-based system (hereafter referred to as the T’ena sensor) to reliably and accurately assess movement quality and efficiency in physically and neurologically healthy adults. Upper limb movement kinematics measured by the T’ena sensor were compared to the gold standard criterion measure during three functional tasks.

## Materials and Methods

### Participants

Ten participants from the San Francisco State University campus (mean age = 27.4, SD = 5.3) participated in the present study. Based on administration of the Revised Edinburgh Handedness Inventory (Dragovic, 2004) which ranks handedness in a battery of common tasks on a scale ranging from −1 (strongly left-handed) to 1 (strongly right-handed), all participants were right handed (mean = 88.7, SD = 7.5). None of the participants had any history of physical or neurological conditions that might interfere with their ability to perform activities of daily living. All participants gave their written informed consent, and the experimental procedures were approved by the San Francisco State University Institutional Review Board committee.

### Instrumentation

**FIGURE 1:**
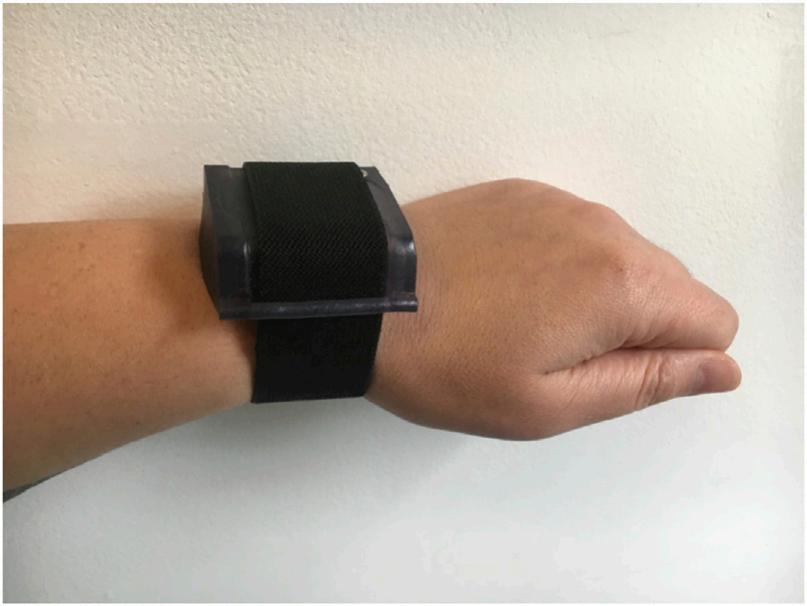
Appearance of the T’ena IMU sensor-based system and approximate mounted position.

Kinematic data were simultaneously obtained with an IMU sensor-based T’ena system placed on the participant’s wrist (Figure 1) and an eight-camera Bonita 10 Vicon optoelectric motion capture system (VICON Motion Systems Ltd., UK). The Vicon system was used as the gold standard reference system, which tracked the three-dimensional position of a 9.5 mm reflective marker attached to the top of the T’ena sensor, and has a temporal and spatial resolution of 100 Hz and 1 mm, respectively.

The T’ena sensor consists of a custom printed circuit board (PCB) that incorporates an ESP32-WROOM32D microcontroller module (Espressif), an ICM20689 IMU sensor (Invensense), and a USB-C connector powered by a 400 mAh lithium-ion battery. The PCB is housed in a stereolithographic (SLA) 3D printed plastic enclosure with dimensions of 50 mm × 7 mm × 20 mm and a total weight of 60 g. The T’ena sensor firmware uses Serial Peripheral Interface (SPI) and a First-In-First-Out (FIFO) buffer to achieve higher efficiency and timing precision with processing and transmission of bulk measurements (Tran et al., 2022). The raw three-axis accelerometer and gyroscope data from the T’ena sensor were used to derive upper limb movement kinematics. Initial calibration of the sensor occurs at the start of each data collection session, and consisted of placing the sensor on a flat table until 1,000 data points were captured. The sampling frequency of the raw data is 100 Hz. During each trial, the raw data were sent from the sensor to the microcontroller *via* the SPI communication protocol, and then to a custom application on a personal computer via classic Bluetooth which saved the incoming data stream in a CSV file for later off-line processing.

### Tasks and Procedure

Kinematic analysis was evaluated through the performance of three tasks commonly used to evaluate post-stroke upper limb function. The Block task is from the grasp subtest of the Action Research Arm Test (Lyle, 1981) and requires the participant grasp a 5 cm^3^ block from the table, place it on the top of a 37-cm-high shelf placed 25 cm away from the proximal edge of the table, and then bring their hand back to rest on the table. The Drink task is from the grip subtest of the Frenchay Arm Test (Heller et al., 1987), and requires participants pick up a soda can, bring the can to their mouth and pantomime taking a sip of the beverage, placing the can on the table, and bringing the hand back to the start position on the table. The Finger to Nose task is from the coordination/speed subtest of the Fugl-Meyer Assessment (Fugl-Meyer et al., 1975), and requires participants bring the tip of the index finger from the side of their body to their nose, and then bring their hand back to against the side of their body. For all trials, participants were instructed to perform the movements at a natural manner at a comfortable speed. Trials performed in a manner that did not coincide with the instructions (e.g., moving prior to verbal start command, placing the object to the wrong target) were repeated immediately.

The order in which the tasks were performed, and the hand used to perform each task, were blocked and counterbalanced across the participants. Participants performed each task 50 times with each hand (dominant, non-dominant), yielding a total of 100 trials. After twenty trials, participants were given a two-minute rest period. The entire experiment lasted approximately 45 min.

### Data Processing

Using Vicon Nexus 2.12 software, the 3D marker position of the reflective marker was reconstructed, and filtered using a Woltring filter, and exported in the CSV file format. Using a custom written MATLAB (The MathWorks®, Version R2021a) scripts, the 3D position data of each axis was transformed into movement velocity using a first-order central difference technique, with the individual vector velocities then summed to derive resultant velocity for each trial.

The raw gyroscope and accelerometer values obtained from the T’ena sensor were processed using proprietary sensor fusion and filtering algorithms written in MATLAB (The MathWorks, Version R2021a). While more details can be found in Hughes et al., 2019, data from each trial were trimmed based on a stationary detection threshold that determines the stationary sections of the beginning and end of the recorded trial, and then excludes the stationary sections from further analysis. Subsequently, the current orientation of the T’ena sensor is calculated using an Attitude Heading Reference System (AHRS) filter, and then transformed from the local sensor coordinate frame to the global (ground) earth frame. To account for gravitational acceleration effects, 1 g was subtracted from the z-axis acceleration, after which velocity for the three axes was derived by taking the integral of the acceleration signals.

Given that the tasks involved all three axes, the individual vector velocities was summed to derive resultant velocity. Movement onset and offset were determined using kinematic criterion, with movement onset calculated as the first instance in the time series when resultant velocity exceeded 1.5% of the first velocity peak, and movement offset calculated as the instance in the time series when the resultant velocity trace dropped, and remained, below 1.5% of the last peak.

**FIGURE 2:**
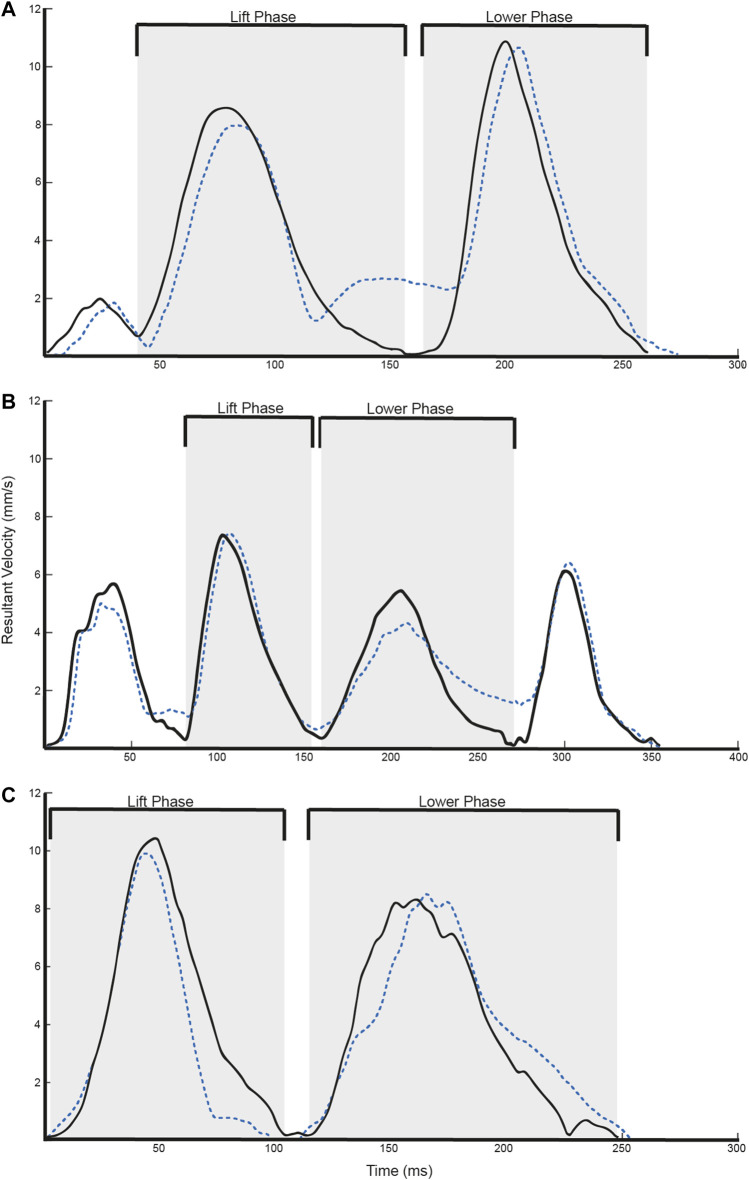
Representative velocity trajectories for the **(A)** Block, **(B)** Drink, and **(C)** Finger to Nose tasks. Solid black lines refer to Vicon data, whereas dotted blue lines refer to T’ena sensor data.

The calculation of the kinematic variables was performed on the time series between movement onset and movement offset. Total movement time was defined as the time period from movement onset to movement offset. Path Length was defined as the total displacement of the hand from the beginning to the end of the movement. In addition, we calculated the peak velocity of the two prominent phases of each task. For the Block task (Figure 2A), the lift phase was defined as the time period between when the block was lifted from the table to the time the block contacted the shelf top. The lower phase was defined as the time period between when the object contacted the shelf top to when the hand was placed on the back on the table. For the Drink task (Figure 2B), it was expected that there would be four peaks in the resultant velocity profile. The lift phase was defined as the time period between when the soda can was grasped to when the soda can reached the participant’s mouth. The lower phase was defined as the time period between when the can left the participant’s mouth to when the can was placed back on the table. For the Finger to Nose task (Figure 2C), two peaks were expected in the resultant velocity time series. The lift phase was defined as the time period between when the hand left the side of the body to when the finger reached the participant’s nose. The lower phase was defined as the time period between when the finger left the participant’s nose to when the hand was placed back to the side of the body.

### Statistical Analysis

First, statistical analysis of the continuous time series data was performed in order to determine the similarity in resultant velocity trajectories between the two systems throughout the task. This was achieved using the regression metrics root mean square error (RMSE), mean absolute error (MAE), and coefficient of determination (*R* ^*2*^). Smaller RMSE and MAE values, and larger *R* ^*2*^ values, indicate higher model precision, indicative of similar spatial trajectories and strong agreement between devices.

Second, statistical analysis was conducted on the: Total Movement Time (ms), Path Length (mm), Peak Velocity of the First Phase (mm/s), and Peak Velocity of the Second Phase (mm/s). For these variables, Pearson product moment correlation coefficients (*r*) were calculated to quantify the degree to which the T’ena sensor and the gold standard Vicon motion capture system are related. Rowntree (1981) conventions were used to interpret coefficients, with values < 0.20 classified as very weak, values between 0.20–0.40 classified as weak, values between 0.40–0.70 classified as moderate, values between 0.70–0.90 as strong, and values > 0.90 as very strong. Intraclass correlation coefficients (ICC 2, 1) were used to evaluate inter-sensor reliability, using the absolute agreement definition between the T’ena sensor and the reference system (Kim, 2013). The strength of the relationship was determined using Evans (1996) empirical classifications, in which values lower than 0.20 are considered very weak, values between 0.20–0.39 are considered weak, values between 0.40–0.59 are considered moderate, values between 0.60–0.79 are considered strong, and values between 0.80–1.0 are considered very strong. In addition to the ICC, the level of agreement between the two systems was calculated using the Bland-Altman method, separately for each variable (Bland and Altman, 1986). Accordingly, differences between the two systems were plotted against the mean of the two devices, thereby providing an indication of potential systemic bias between the devices (i.e., mean bias). The level of significance was set to 0.05, and all statistical analysis was performed using the R environment (RStudio version 1.4.1103, RStudio, Boston, MA).

## Results

Overall, 3,000 trials were simultaneously obtained by the T’ena sensor and a Vicon motion capture system (10 participants × 2 hands × 3 tasks × 50 trials). As can be seen in Figures 2A–C, the T’ena sensor produced resultant velocity trajectories that were similar to that captured by the gold-standard Vicon motion capture system. The similarity in kinematic waveforms was confirmed by the regression metrics RMSE, MAE and *R* ^*2*^ values between the predicted and true velocity profile (Table 1). Considering all tasks, the RMSE, MAE, and *R* ^*2*^ values ranged from 1.24 to 2.70, 1.00 to 2.26, and 0.734 to 0.891 respectively.

**TABLE 1:** Root mean square error (RMSE), mean absolute error (MAE), and coefficient of determination (*R* ^*2*^) of the T’ena system to the ground-truth Vicon motion capture system.

	RMSE	MAE	*R* ^*2*^
Block	1.47	2.26	0.891
Drink	1.24	1.00	0.834
Finger to Nose	2.70	2.13	0.734

RMSE, root mean square error; MAE, mean absolute error; *R* ^*2*^, coefficient of determination.

Mean (SD) values estimated by the T’ena sensor and Vicon system is provided in Table 2. Overall, strong to very strong agreements (based on Rowntree, 1981 conventions) were observed for all metrics across the Block task with Pearson product moment correlation coefficient (*r*) values of 0.842–0.947. Correlation coefficients were also high for all Drink task metrics (*r* = 0.845–0.891) except path length (*r* = 0.721). The lowest correlations between devices was found for the Finger to Nose task (*r* = 0.648–0.894), with strong correlations found for movement time (*r* = 0.731) and lift phase peak velocity (*r* = 0.894), and moderate correlations for path length (*r* = 0.648) and lower phase peak velocity (*r* = 0.680).

**TABLE 2:** Kinematic metrics, correlations, intraclass correlations, and Bland-Altman analysis indicating bias and limits of agreement between the T’ena sensor and Vicon motion capture system.

	Vicon Mean (SD)	T’ena Mean (SD)	ICC(2,1)	Pearson’s *r*	Mean difference	Lower limit of agreement	Upper limit of agreement
Block							
Movement Time	2,598.10 (327.47)	2,638.05 (333.53)	0.836 (0.808–0.860)*	0.842*	−40.49	−403.90	322.88
Path Length	1,174.89 (143.43)	1,197.60 (155.29)	0.932 (0.883–0.956)*	0.947*	−23.58	−122.09	74.92
Lift Phase Peak Velocity	11.19 (1.45)	11.10 (1.45)	0.887 (0.871–0.900)*	0.888*	0.09	−1.25	1.44
Lower Phase Peak Velocity	10.84 (1.27)	11.53 (1.53)	0.773 (0.769–0.901)*	0.882*	−0.69	−2.11	0.73
Drink							
Movement Time	3,683.15 (368.39)	3,761.05 (340.77)	0.809 (0.729–0.860)*	0.846*	−77.89	−492.89	337.10
Path Length	1,068.43 (139.47)	1,117.74 (165.69)	0.675 (0.555–0.756)*	0.721*	−49.31	−278.00	179.39
Lift Phase Peak Velocity	7.30 (1.70)	6.96 (1.16)	0.852 (0.710–0.912)*	0.891*	0.34	−0.83	1.51
Lower Phase Peak Velocity	7.31 (1.70)	6.50 (1.23)	0.702 (0.259–0.853)*	0.845*	0.80	−1.02	2.62
Finger to Nose							
Movement Time	2,604.07 (349.42)	2,543.15 (333.80)	0.719 (0.672–0.758)*	0.731*	60.29	−431.17	553.00
Path Length	1,058.28 (103.31)	1,105.43 (208.74)	0.496 (0.420–0.561)*	0.648*	−47.15	−364.86	270.57
Lift Phase Peak Velocity	11.56 (1.85)	12.77 (2.01)	0.717 (-0.007-0.895)*	0.894*	−1.21	−3.01	0.59
Lower Phase Peak Velocity	8.90 (1.58)	9.46 (2.11)	0.530 (0.209–0.687)*	0.680*	−0.56	−3.74	2.63

* refers to statistical significance < 0.05.

Inter-sensor reliability values (ICC 2, 1) are presented in column 4 of Table 2. Regarding all tasks and metrics, the data obtained by the T’ena system were associated with the data obtained by VICON (ICC = 0.496–0.932). Inter-sensor reliability for the Block task yielded very strong ICCs for all metrics (ICC = 0.836–0.932) except lower phase peak velocity (ICC = 0.773). For the Drink Task, very strong ICC values were found for movement time and lift phase peak velocity (ICC = 0.809 and 0.852), and strong ICC values were found for path length and lower phase peak velocity (ICCs = 0.675 and 0.702). The lowest reliability was found for the Finger to Nose task, with moderate ICCs observed for path length and lower phase peak velocity (ICCs = 0.496 and 0.530) and strong ICCs for movement time and lift phase peak velocity (ICCs = 0.719 and 0.717).

Bland Altman analysis was conducted to evaluate the limits of agreement between the T’ena sensor and Vicon data for all tasks and kinematic metrics (see Supplementary Material and Table 2 columns 6–8). Mean differences between the two methods of measurement (i.e., the bias) indicated whether the T’ena sensor was overestimating (positive bias) or underestimating (negative bias) behavior compared with kinematics obtained from the Vicon system. The Bland Altman analysis revealed that the T’ena sensor underestimated movement time for the Block (mean difference = −40.49) and Drink tasks (mean difference = −77.89 ms), but overestimated movement time for the Finger to Nose task (mean difference = 60.29 ms). The path length data displayed underestimation biases for all three tasks, with lower mean difference for the Block task (−23.58 mm) compared to the Drink and Finger to Nose tasks (−49.31 mm and −47.15 mm). The Bland Altman analysis for first phase peak velocity (mean difference = −1.21 and 0.34) and second phase peak velocity (mean difference = −0.69 and 0.80) revealed a high level of agreement between actual peak velocity obtained by the Vicon system and peak velocity calculated by the T’ena sensor across all three tasks.

## Discussion

The aim of the present study was to determine the ability of the T’ena IMU sensor-based system to reliably and accurately assess movement quality and efficiency in three ADL tasks commonly used to evaluate post-stroke upper limb function. Analysis of the resultant velocity profile demonstrated excellent correspondence between the T’ena system and the ground-truth Vicon motion capture system was strong, with strong to very strong validity values across all tasks and metrics. Moreover, the RMSE, MAE, and *R* ^*2*^ values obtained in the current study are congruent and similar to prior studies that have recorded movement kinematics using wearable systems with multiple IMUs (e.g., Cho et al., 2018; Teufl et al., 2019). Taken together, these results indicate that the T’ena system was capable of reliably measuring movement kinematics in clinically relevant movements. The implications of these results for the transition of this technology from the research lab to the marketplace to the clinic are discussed below.

The largest differences between T’ena IMU sensor-based system and the Vicon motion capture system derived movement kinematics were observed for the Nose to Finger task, which exhibited larger RMSE and lower *R* ^*2*^ values than the Block and Glass tasks. In addition, kinematic metrics of the Finger to Nose task yielded lower Pearson’s *r* values than the other tested tasks. Bland-Altman analysis revealed that these differences were largely systematic in nature with movement time values derived from the T’ena system that were 60 ms longer than those derived from the ground-truth Vicon system. The difference in movement time is likely a result of the required end posture of the tasks. For the Block and Glass tasks, the hand was to be placed back on the table at the end of the task, whereas participants ended the task by bringing their hand back to the side of the body for the Nose to Finger task. In the former two tasks, the firm surface serves to brake the movement and minimizes small changes in Vicon marker displacement upon contact with the table (Figures 2A,B). In contrast, because there is some compliance in the thigh structure (e.g., skin, adipose tissue), the hand rebounds against the thigh at the end of the movement, which is evident as a small velocity curve at the end of the movement (Figure 2C). In light of these findings, it would seem sensible to verify the calculation of movement offset by synchronizing both the Vicon and T’ena systems with a mechanical target switch that can send a 5 v pulse to the Vicon system when the hand leaves and contacts the target.

Aligning with previous studies (Hughes et al., 2019a; Hughes et al., 2019b), we have shown promising validity for movement kinematics detected using single IMU-based systems. Although increasing the number of IMUs would allow for the calculation of kinematics across multiple joints, it would also increase the cost of the system as well as its usability as a decentralized rehabilitation tool. For example, donning and doffing wearable sensors might be challenging for patients with poor distal arm function or high spasticity. While inconvenient for physically abled individuals, such experiences have a greater impact for people with neurological and physical disabilities in that it can reduce motivation and engagement with the device, as well as commitment to their rehabilitation protocol.

### Further Research

The T’ena sensor provides more objective measurements of movement kinematics than conventional clinical assessments, which clinicians can use to administer individualized rehabilitation protocols as well as adjust therapy plans depending on patient progress. While a useful tool for any rehabilitation professional, the ability to use clinically relevant information about movement quality and efficiency is especially helpful for rehabilitation clinicians with limited knowledge and experience treating patients with stroke or other physical or neurological disorders. On the basis of this study, there are three key areas to consider for future development of the T’ena sensor system.

First, given that the focus was on revalidating the T’ena sensor after the considerable changes to the hardware and firmware of the system, the current study included only neurologically and physically healthy individuals. The next step in this work is to examine the ability of the T’ena sensor to discriminate between arm impairment in varying pathological populations, and to determine the extent to which the sensor-based metrics correlate to validated condition-specific assessment scales.

Second, as we expand our validation work, efforts will be focused on determining the types of tasks that the T’ena sensor can accurately quantify. In this study we have shown reduced performance of the T’ena algorithm to derive accurate movement kinematics during the Finger to Nose task, and will explore whether we can increase the accuracy of the signal processing algorithm by tuning the parameters used to detect stationary states and IMU orientation, as well as exploring whether deep learning models trained using the ground truth motion capture data can improve the ability of the T’ena sensor to accurately measure movement kinematics. In addition, due to the fact IMU-based systems often suffer from errors that accumulate over time (Geiger et al., 2008), we will first focus on shorter duration tasks from clinically validated measures (e.g., “turning the key in lock” from the Wolf Motor Function test, “shoulder abduction” from the Fugl-Meyer Assessment Upper Extremity) before turning our attention to longer duration (e.g., the 6-minute Walk test, cf. Storm et al., 2020) and repetitive actions (e.g., the Box and Blocks test, cf. Mathiowetz et al., 1985) that will likely not yield accurate enough metrics using the current algorithm.

Third, the T’ena sensor will be incorporated into a tele-rehabilitation application that runs on the Android platform (Hughes et al., 2019c), after which participatory iterative design approaches will be used to develop a robust system that can be used in medically unserved areas. As a research group, we are particularly interested in supporting the provision of rehabilitative care for persons living in countries exhibiting fragile and conflict-affected settings where the destruction of transportation and infrastructure, emigration of healthcare workers, and financing of health systems lead to a substantial degradation in the ability to deliver in-person health services (Bornemisza et al., 2010). According to the most recent data from The World Bank, Data (2022), there are 39 countries and territories that are affected by violent conflict or institutional and social fragility. Of these fragile and conflict-affected countries, 50% are classified as having low-income economies, and 31.6% are classified as having lower-middle-income economies (The World Bank, Data, 2022), which together account for 880 million people, or 11.10% of the world’s population. That said, technology-enabled solutions that are well-planned and consider the context-specific barriers (e.g., geographical accessibility, public acceptability of services, adequacy of infrastructure, attacks on healthcare facilities) can make a significant impact on the health needs of the target population (Saleh et al., 2018; Bowsher et al., 2021).

## Conclusion

The purpose of the current experiment was to ascertain whether the T’ena sensor can reliably and accurately assess movement quality and efficiency in physically and neurologically healthy adults. Overall, the results indicated that the T’ena sensor and the gold standard motion capture system were significantly correlated for all tasks and measures (*r* range = 0.648–0.947), with RMSE, MAE, and *R* ^*2*^ values ranging from 1.24 to 2.70, 1.00 to 2.26, and 0.734 to 0.891 respectively. Taken together, the research presented indicates that the T’ena single IMU sensor system is a valid and objective method by which to measure movement kinematics during functional tasks. Context-appropriate enabling technologies specifically designed to address barriers to quality health services in LMICs (such as the T’ena system) can promote high-quality rehabilitative services for individuals living in vulnerable settings and medically underserved areas while also accelerating progress towards the United Nations Sustainable Development Goal 3.

## Data Availability

The raw data supporting the conclusions of this article will be made available by the authors, without undue reservation.
