# Supplementary material for: Accuracy and Validity of a Single Inertial Measurement Unit-Based System to Determine Upper Limb Kinematics for Medically Underserved Populations
Source: Front Bioeng Biotechnol. 2022 Jun 27;10:918617. doi: 10.3389/fbioe.2022.918617 (PMC9271671; doi:10.3389/fbioe.2022.918617)
Supplement: Supplementary file 1 [file DataSheet1.docx]

**Bland Altman Plots for the Block Task**

**
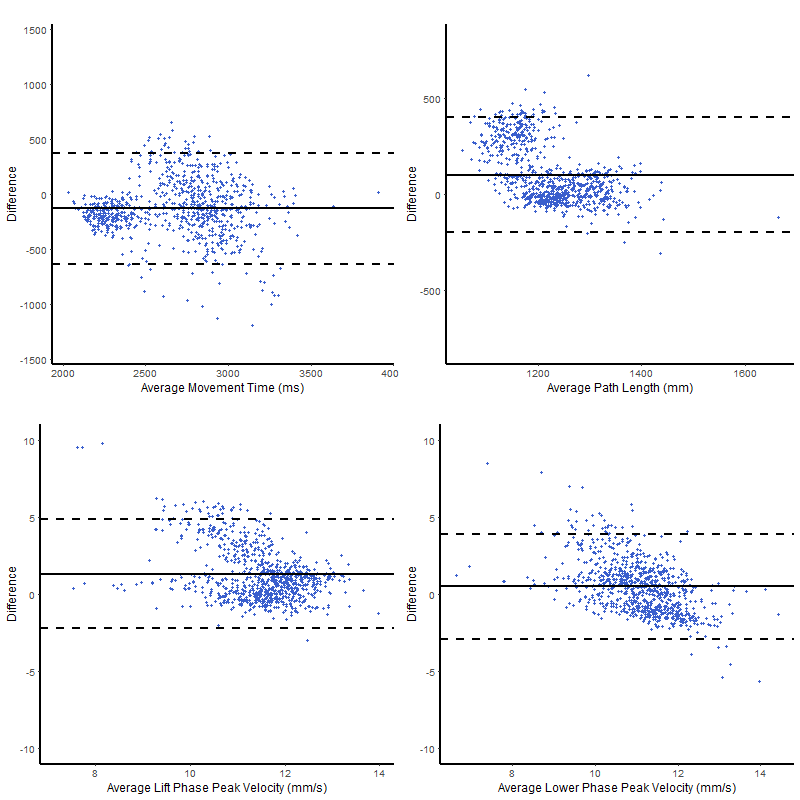
**

**Bland Altman Plots for the Glass Task**

**
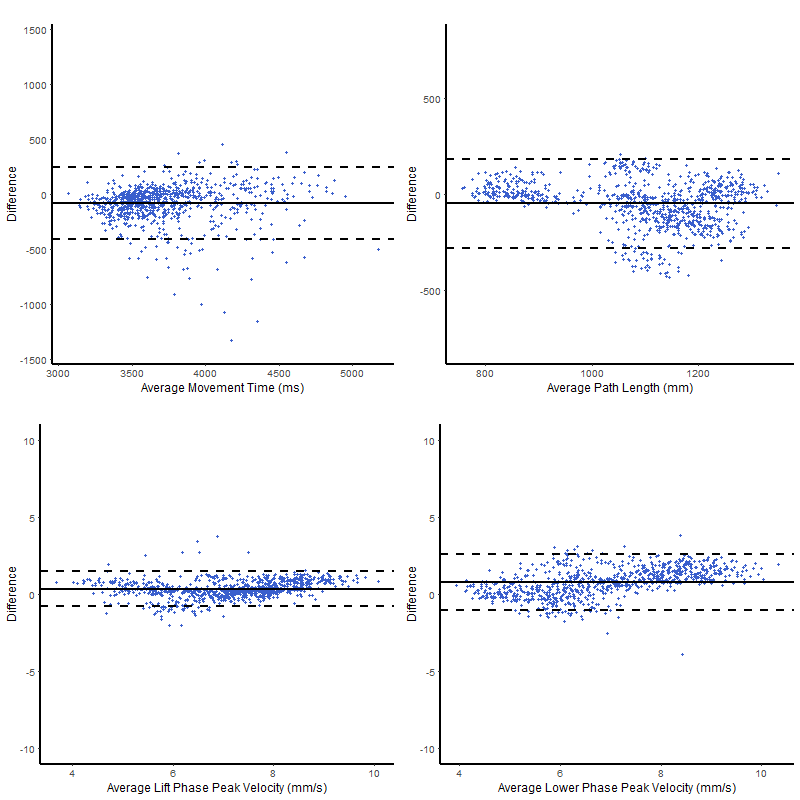
**

**Bland Altman Plots for the Finger to Nose Task**

**
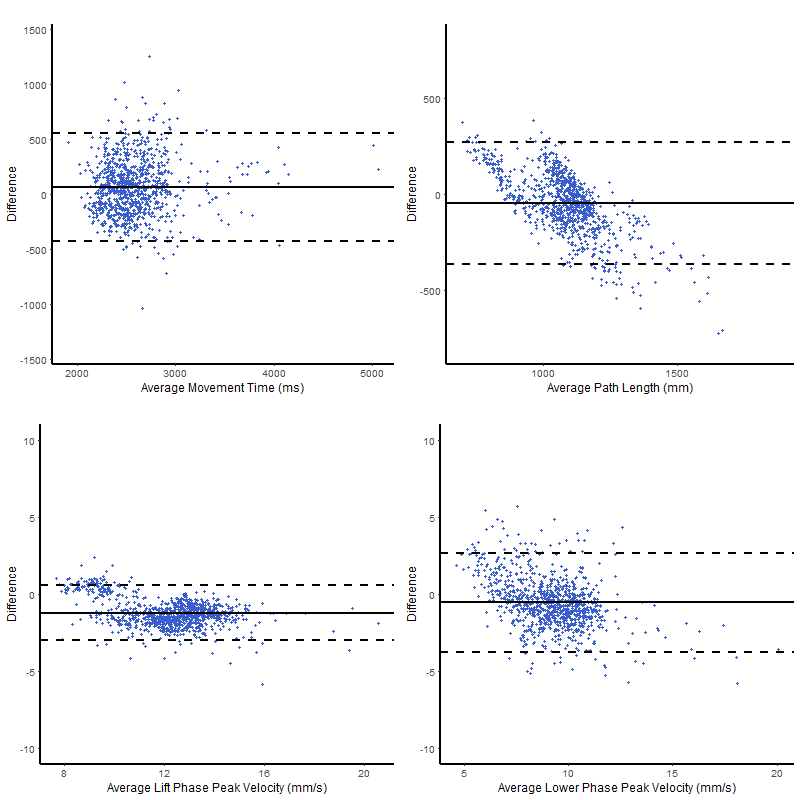
**
